# Loss of HTLV-1–specific CD8^+^ T-cell immunity in virus carriers predisposed to adult T-cell leukemia/lymphoma

**DOI:** 10.1016/j.bneo.2026.100235

**Published:** 2026-04-27

**Authors:** Devon A. Weterings, Lisa Lam Chiou Yee, Patricia Watber, Jerico Baylon, Claire Greiller, Graham P. Taylor, Lucy B. M. Cook, Aileen G. Rowan

**Affiliations:** 1 Department of Infectious Disease, Faculty of Medicine, Imperial College London, London, United Kingdom; 2 National Centre for Human Retrovirology, Imperial College Healthcare NHS Trust, London, United Kingdom; 3 Department of Haematology, Imperial College Healthcare NHS Trust, London, United Kingdom

## Abstract

Adult T-cell leukemia/lymphoma (ATL) is caused by chronic infection with human T-lymphotropic virus type 1 (HTLV-1). HTLV-1 contains highly immunogenic CD8**^+^** T-cell epitopes that elicit high frequencies of virus-specific CD8**^+^** T cells in most virus carriers. Despite the virus being present in the tumor, HTLV-1–specific CD8**^+^** cells are often undetectable in ATL. To characterize HTLV-1–specific CD8**^+^** T cells during ATL development, we studied a subgroup of people living with asymptomatic HTLV-1 infection at very high risk of developing ATL. These so-called “high-risk” carriers have suspected premalignant lesions: expanded, HTLV-1–infected “ATL-like” clones circulating in their peripheral blood. Compared to viral antigen–burden matched controls, high-risk carriers had significantly fewer Tax-specific IFN-γ**^+^**CD8**^+^** cells in peripheral blood. Furthermore, *ex vivo* CD8**^+^** T cells from high-risk carriers did not efficiently kill autologous HTLV-1–infected T cells, including premalignant ATL-like clones. We stained Tax_11–19_/HLA-A∗0201 pentamer**^+^**CD8**^+^** T cells to test whether the low frequencies of functional CD8+ T cells resulted from phenotype or absolute frequency of HTLV-1–specific CD8**^+^** T cells. High-risk carriers had significantly lower frequencies of Tax_11–19_/HLA-A∗0201 pentamer**^+^**CD8**^+^** T cells than controls, but we observed no difference in effector function, memory phenotype, or expression of checkpoint control molecules. In contrast, there was no difference in the frequency of CD8**^+^** T cells specific for other viruses (cytomegalovirus, Epstein-Barr virus, influenza virus) between high-risk carriers and controls. This is the first report of HTLV-1–specific immune dysregulation in the premalignant stage of ATL. Low frequencies of HTLV-1–specific CD8**^+^** T cells may contribute to ATL development and may be a novel therapeutic target for ATL prevention.

## Key Points

• HTLV-1 carriers at high risk of ATL have low frequencies of circulating HTLV-1–specific CD8**^+^** T cells, suggesting immune dysregulation.
• Low frequencies of HTLV-1–specific CD8^+^ T cells may contribute to ATL development, suggesting a potential novel target for ATL prevention.

## Visual Abstract

**Figure:**
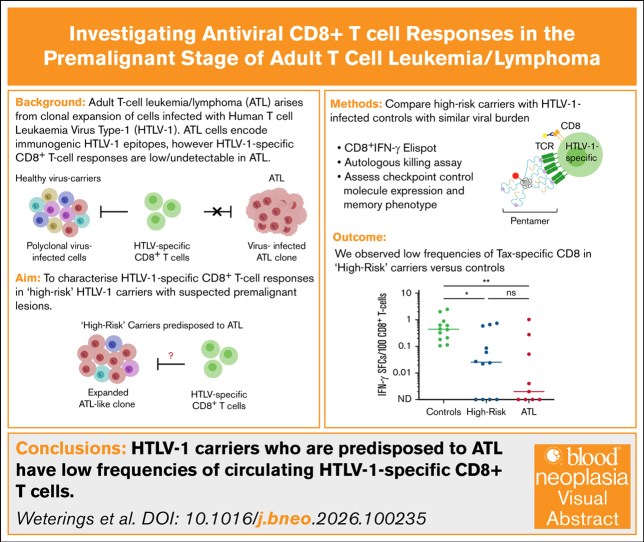

## Introduction

Adult T-cell leukemia/lymphoma (ATL) is an aggressive CD4**^+^** T-cell malignancy caused by human T-lymphotropic virus type 1 (HTLV-1). ATL usually develops decades after initial infection in people living with HTLV-1 with high proviral load (PVL; >4%, percentage of peripheral blood mononuclear cells [PBMCs] carrying integrated HTLV-1 proviruses). ATL is classified by the World Health Organization classification into indolent subtypes (chronic and smoldering) and aggressive types (acute and lymphoma).^1^^,^^2^ Aggressive ATL has an overall survival of 8 to 10 months,^3^ and ∼50% of indolent subtypes transform to aggressive ATL within 2 years after diagnosis.^4^ Treatment outcomes are poor; hence, there is an urgent need for novel interventions.

HTLV-1 primarily infects CD4**^+^** T cells and integrates a DNA copy of the 9-kb viral genome (provirus) into the host cell genome. The virus persists in the host by driving mitotic proliferation of infected T cells via expression of viral proteins such as Tax and HBZ, leading to the establishment of thousands of distinct HTLV-1–infected T-cell clones.^5^ Each clone is characterized by the unique provirus integration site in the host genome and the T-cell receptor expressed by the clone.^6^^,^^7^ Viral protein expression elicits a cytotoxic T-lymphocyte (CTL) response, and most HTLV-1 carriers generate high frequencies of chronically activated HTLV-1–specific CTLs. An efficient CTL response to HTLV-1 has been reported to play an important role in limiting PVL and risk of associated diseases.^8^ The viral protein Tax is immunodominant for CD8**^+^** T cells, and a high frequency of Tax-specific CD8**^+^** T cells can be detected in most HTLV-1 carriers.^9^ Additionally, a detectable response to HBZ is associated with low risk of developing HTLV-1–related disease.^10^

In >90% of ATL cases, the malignant tissue consists of a single HTLV-1–infected T-cell clone.^11^^,^^12^ Patients with ATL are immunocompromised^13^ and have low frequency, diversity, and function of HTLV-1–specific CTLs.^14^ There is also evidence that ATL clones evolve to escape the immune response. In over 50% of ATL cases, expression of Tax protein is lost in the ATL clone by mutation, deletion, or methylation.^15^ The malignant clone also carries mutations in the major histocompatibility complex (MHC) class I genes (*HLA-A* and *HLA-B*), and mutations are found in other genes involved in immune surveillance, such as CD58, FAS, and PD-L1.^16^^,^^17^

We recently reported that premalignant HTLV-1–infected cells (“ATL-like clones”) circulate in the blood up to 10 years before onset of ATL.^18^ Oligoclonal expansion of HTLV-1–infected cells can be detected by flow cytometric analysis of TCRVβ subunit expression by T cells that carry HTLV-1 proviruses (CCR4**^+^**CADM1**^+^**CD26**^−^**CD4**^+^** T cells).^7^^,^^19^, ^20^, ^21^ Oligoclonality scores, calculated from the frequency distribution of TCRVβ subunits, can be used to compare individuals. A score of >0.770 can be used to identify individuals with putative premalignant lesions—circulating ATL-like clones^22^—who are therefore considered at high risk of developing ATL (“high-risk carriers”).

Little is known about the CD8**^+^** T-cell response during the development of ATL. Here, we characterized Tax expression and HTLV-1–specific CTL responses in high-risk carriers. We demonstrate that despite having no symptoms of malignancy, high-risk carriers share many features of ATL: 50% of ATL-like clones circulating in high-risk carriers do not express the CTL-immunodominant antigen Tax, and high-risk carriers have significantly lower frequencies of Tax-specific CD8**^+^** T cells than antigen-load–matched controls.

## Materials and methods

### Patient selection

People living with HTLV-1 attending the National Centre for Human Retrovirology (Imperial College Healthcare NHS Trust, St Mary’s Hospital, London) donated blood for research after providing written informed consent. Research was conducted under the governance of the Communicable Diseases Research Group Tissue Bank, approved by the UK National Research Ethics Service (09/H0606/106, 15/SC/0089, 20/SC/0226, 25/SC/0209).

A group of “high-risk carriers,” with circulating abnormally expanded ATL-like clones but no clinical evidence (signs, symptoms, and laboratory markers) of malignancy, was identified based on an oligoclonality score >0.770 and a PVL >4%^22^ (supplemental Table 1). The comparator group comprised HTLV-1 carriers with an oligoclonality score <0.770 and a PVL >4%. Patients with ATL were included as positive controls for comparison where possible.

### PBMC isolation

PBMCs were isolated from whole blood by density-gradient centrifugation using Histopaque-1077 (Sigma-Aldrich) from EDTA-anticoagulated blood. Isolated PBMCs were washed twice in phosphate-buffered saline (PBS), then cryopreserved in fetal bovine serum (FBS; Gibco) with 10% dimethyl sulfoxide (Sigma) and stored at −150°C until used.

### PVL and oligoclonality score quantification

PVL and oligoclonality score (OCI-flow) were assayed in the Molecular Diagnostic Unit (Imperial College London) as part of routine clinical investigation, as previously described.^22^^,^^23^ To assess oligoclonality, PBMCs were stained with antibodies specific for CD3, CD4, CD8, CCR4, CD26, and 24 TCRVβ subunits (IOTest Beta Mark TCRVbeta Repertoire Kit, Beckman Coulter) and analyzed by flow cytometry. The frequencies of the 25 subsets of CD3**^+^**CD4**^+^**CCR4**^+^**CD26^−^cells (24 subsets expressing known TCRVβ subunits and 1 subset expressing off-panel subunits) and 25 subsets of CD3**^+^**CD8**^+^**CCR4**^+^**CD26^−^ cells were quantified. These frequencies were used to calculate the oligoclonality score,^24^ derived from the Gini Index.^25^

### CD8**^+^** T-cell killing assay

The protocol was detailed previously.^7^ CD8**^+^** T cells were isolated from cryopreserved PBMCs by positive selection using magnetic beads (Miltenyi Biotec) per manufacturer’s protocol. Freshly isolated autologous *ex vivo* CD8**^+^** cells and CD8-depleted PBMCs of each donor were mixed at a range of effector-to-target ratios: CD8-depleted, physiological CD8**^+^**:CD4**^+^** ratio 1:1, and 3:1 in duplicate. Cells were cocultured for 18 hours at 37°C and 5% CO_2_ in RPMI containing 10% FBS, 50 U/mL penicillin, 50 μg/mL streptomycin, 2 mM L-glutamine (Gibco), and 20 μg/mL DNase (Sigma).

After incubation, cells were stained for 5 minutes with 1 μL/mL Zombie Near-Infrared stain (BioLegend). Cells were washed with PBS 10% FBS and incubated for 20 minutes at room temperature (RT) with antibodies specific for CD3, CD4, CD8, CADM1, and TCRVβ (supplemental Table 4), using an anti-TCRVβ antibody specific for the subunit expressed by the dominant clone. Biotinylated antibodies were detected by staining with streptavidin-BV421 (BioLegend) for 10 minutes at RT in PBS 10% FBS. Cells were fixed and permeabilized using FoxP3 staining buffers (eBioscience) and stained with anti-Tax antibody for 30 minutes at RT in permeabilization buffer. Cells were washed with permeabilization buffer and stored in PBS 10% FBS at 4°C until analysis. Data were acquired using a BD LSRFortessa and analyzed using the Kaluza software (gating strategy in supplemental Figure 3).

### Analysis of CD8**^+^** T-cell killing assay

The frequency of cells in each population was obtained using the gating strategy in supplemental Figure 3. The rate at which cells in a given population were killed by autologous CD8**^+^** T cells (percentage of cells killed per percentage of CD8**^+^** cells per day) was estimated for each donor as described in Asquith et al^26^ using the following equation: $\frac{dy}{dt}=c-\varepsilon yz$, where y is the proportion of target cells in total CD4**^+^** cells; c is the rate of Tax expression, which is assumed to be constant during short-term culture; ε is the CD8**^+^** cell–mediated lytic efficiency; and z is the proportion of CD8**^+^** in total CD3**^+^** cells. The model was solved analytically and fitted to cell survival flow cytometry data using SPSS v28, providing an estimate of lytic efficiency (ε) in each individual.

### Long-range PCR

Long-range polymerase chain reaction (PCR) was performed using the UltraRun LongRange PCR Kit (Qiagen). A reaction mixture with a final concentration of 1× master mix (4×), 0.5μM each of the forward and reverse primers (supplemental Table 2) and template DNA (1000 proviruses), was prepared and amplified using the cycling conditions detailed in supplemental Table 3. PCR products were electrophoresed in 0.8% agarose gel and visualized under UV light.

### ELISpot assay

Cryopreserved PBMCs were depleted of CD4**^+^** T cells by positive selection using magnetic beads (Miltenyi Biotec), per manufacturer’s protocol. CD4-depleted cells were cultured in duplicate at a density of 100 000 cells per well at 37°C for 6 hours with pools of 5μM overlapping 15-mer peptides (offset by 4 amino acids) spanning Tax or HBZ, or medium alone. CD4-depleted cells were also cultured in the presence of a pool of 32 peptides derived from cytomegalovirus (CMV), Epstein-Barr virus (EBV), and influenza virus (PepTivator CEF MHC Class I Plus Peptide Pool; Miltenyi Biotec) at a concentration of 0.6μM. For the HLA-A∗0201**^+^** cohort, PBMCs were cultured in duplicate at a density of 100 000 cells per well at 37°C for 6 hours in the presence of 5μM HTLV-1 Tax_11-19_ peptide or 5μM CMV pp65_495-503_ peptide, or medium alone.

After 6-hour incubation, IFN-γ**^+^** cells were quantified by ELISpot (Mabtech) following the manufacturer’s instructions. Spot-forming cells were counted using an automated ELISpot reader (Autoimmun Diagnostika GmbH). Positive responses were defined as greater than the mean plus 2 standard deviations of the number of spots in the medium-only control.

### Pentamer staining

Cryopreserved PBMCs were washed once with PBS 10% FBS and incubated for 10 minutes at RT with 10% Fc Block in PBS 10% FBS. The HTLV Tax_11-19_/HLA-A∗0201 and CMV pp65_495-503_/HLA-A∗0201 pentamers were directly added and incubated for another 10 minutes at RT. Cells were washed once with PBS 10% FBS and stained for 5 minutes with 1 μL/mL Zombie stain (BioLegend). Cells were then washed once with PBS 10% FBS and incubated for 20 minutes at RT with antibodies specific for CD14, CD19, CD3, and CD8. Cells were also stained with antibodies specific for CTLA-4, TIM-3, TIGIT, LAG-3, and PD-1 in the checkpoint protein panel or for CCR7, CD95, and CD45RA in the memory phenotype panel (supplemental Table 4). Cells were fixed with Fixation Buffer (BioLegend) and stored at 4°C until acquisition. Data were acquired using a BD LSRFortessa and analyzed using the Kaluza software (gating strategy outlined in supplemental Figures 4-5 and 7). LAG-3, TIM-3, and surface CTLA-4 can be detected using the checkpoint staining panel (supplemental Figure 6).

### Statistical analysis

Statistical analysis was carried out using GraphPad Prism 10 v10.4.1 (GraphPad Software).

## Results

### Expression of Tax protein by ATL and ATL-like clones

To begin, we assessed expression of the master regulator of viral transcription and immunodominant CTL antigen Tax in high-risk carriers (n = 12), PVL-matched controls (n = 12), and patients with ATL (n = 9) (supplemental Table 1) after short in vitro culture of CD8-depleted PBMCs.

**Figure 1.**
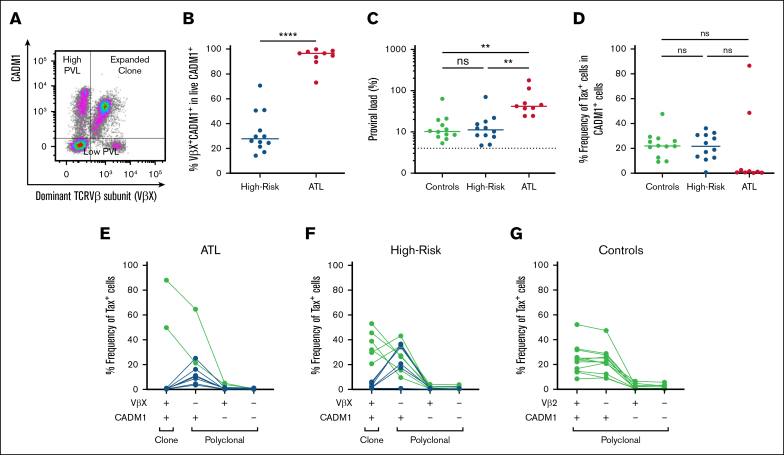
**Frequency of Tax-expressing CADM1^+^CD4^+^ T cells.** CD8**^+^** cell–depleted PBMCs of high-risk carriers (n = 12), controls with matched PVLs (n = 12), and patients with ATL (n = 9) (supplemental Table 1) were cultured for 18 hours and stained for surface markers along with intracellular Tax expression. (A) Representative flow cytometry plot of total live CD3**^+^**CD4**^+^** cells from a high-risk carrier, showing the cell populations identified. ATL and ATL-like clones were identified as VβX**^+^**CADM1**^+^** cells, high-PVL nonclonal cells were identified as VβX^−^CADM1**^+^** cells, and low-PVL cells were identified as VβX**^+^**CADM1**^−^** cells and VβX^−^CADM1**^−^** cells. (B) Frequency of the abnormally expanded clone (VβX**^+^**CADM1**^+^** cells) in total live HTLV-1–infected cells (CADM1**^+^** cells). (C) PVL in each group. All patients selected in this study had a PVL >4% (marked as dotted line). (D) Frequency of Tax-expressing HTLV-1–infected cells (Tax**^+^**CADM1**^+^** cells) in total live HTLV-1–infected cells (CADM1**^+^** cells) was quantified in each group. (E-G) Frequency of Tax-expressing cells. Cells were gated on live CD3**^+^**CD4**^+^** cells that were positive or negative for CADM1 and the dominant TCRVβX as indicated. In controls, who do not have a detectable ATL-like clone, cells were stained for the most frequently utilized TCRVβ (Vβ2). Tax**^+^** ATL and ATL-like clones are plotted in green, and Tax^low^ ATL and ATL-like clones are plotted in blue. Horizontal line denotes median in panels B-D. Statistical analysis: (B) Mann-Whitney, 2-tailed, 95% confidence interval; (C-D) Kruskal-Wallis test with Dunn post-test, 95% confidence interval. ns denotes *P* > .05; ∗∗, *P* < .01 and ∗∗∗∗, *P* < .0001.

T cells that carried HTLV-1 were identified by CADM1 expression.^7^^,^^19^ Where present, expanded clones were identified by staining the TCRVβ subunit expressed by the expanded clone (VβX). Thus, VβX**^−^**CADM1**^+^** cells identified high-PVL nonclonal cells, VβX**^+^**CADM1**^+^** cells identified ATL or ATL-like clones, and VβX**^+/−^**CADM1**^−^** cells identified largely uninfected cells (Figure 1A). Samples from controls were stained for the most frequently utilized TCRVβ (Vβ2); hence, Vβ2**^+^**CADM1**^+^** and Vβ2**^−^**CADM1**^+^** cells are both high-PVL nonclonal cell populations. In patients with ATL, the frequency of VβX**^+^**CADM1**^+^** cells ranged from 73.01% to 99.75% (median = 96.48%) of CADM1**^+^** cells (Figure 1B). In high-risk carriers, the ATL-like clones were present at significantly lower frequencies, ranging from 14.16% to 70.56% (median = 27.77%) of CADM1**^+^** cells. High-risk carriers and controls have, by experimental design, a similar antigen burden, median PVL (10.25% vs 11.20%), and median frequency of Tax**^+^**CADM1**^+^** cells (21.95% vs 21.66% of CADM1**^+^** cells; Figure 1C-D).

The frequency of Tax-expressing cells was quantified in each group (Figure 1E-G). As expected, Tax expression was higher in CADM1**^+^** cells compared to CADM1**^−^** cells in all 3 groups, because CADM1**^−^** cells are mostly uninfected cells.^19^ In patients with ATL, the frequency of Tax**^+^**VβX**^+^**CADM1**^+^** cells ranged from 0.21% to 87.91% (median = 0.34%; Figure 1E). In high-risk carriers, the frequency of Tax**^+^**VβX**^+^**CADM1**^+^** cells ranged from 0.77% to 52.89% (median = 13.24%; Figure 1F). In the controls, the frequency of Tax**^+^** cells was similar in Vβ2**^+^**CADM1**^+^** and Vβ2**^-^**CADM1**^+^** cells, ranging from 8.51% to 52.21% (median = 23.76%) and from 8.93% to 47.37% (median = 21.76%), respectively (Figure 1G).

ATL and ATL-like clones were classified as either Tax^low^ (<5% Tax**^+^** cells in VβX**^+^**CADM1**^+^** cells) or Tax**^+^** (>5%). Seven of 9 (78%) patients with ATL had a Tax^low^ ATL clone, whereas 2 (22%) had a Tax**^+^** ATL clone (Figure 1E). In the high-risk carrier group, 50% (6/12) had a Tax^low^ ATL-like clone and 50% (6/12) had a Tax**^+^** ATL-like clone (Figure 1F). In addition, using long-range PCR, it was identified that 50% (3/6) of Tax^low^ ATL-like clones had deletions in the provirus (supplemental Figure 1).

These results demonstrate, in the cohort tested, that 50% of ATL-like clones do not express Tax protein.

### CD8**^+^** T-cell killing of HTLV-1-infected cells

**Figure 2.**
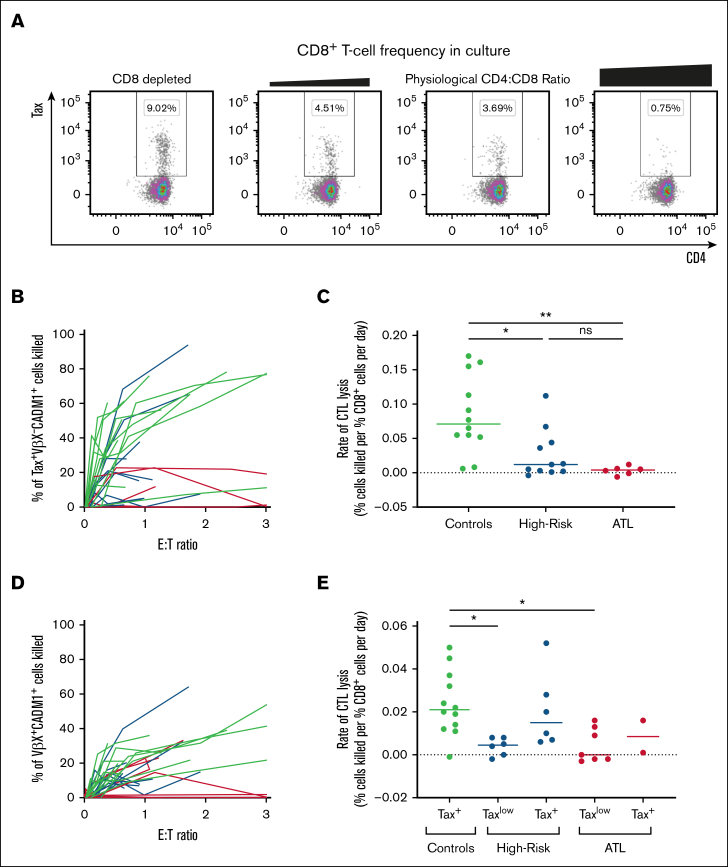
**Rate of lysis by *ex vivo* autologous CD8^+^ T cells.** CD8**^+^** cell–depleted PBMCs were incubated for 18 hours either alone or in the presence autologous CD8**^+^** T cells at a range of ratios. (A) Representative data of CD8**^+^** T-cell–mediated lysis. Cells are gated on live CD3**^+^**CD4**^+^** T cells in 4 dot plots with progressively increasing numbers of autologous CD8**^+^** T cells. The numbers in each quadrant indicate the percentage of CD4**^+^** T cells that express Tax. (B) The proportion of Tax**^+^**VβX**^-^**CADM1**^+^** cells killed with varying CD8**^+^** T-cells-to-target ratios was measured in high-risk carriers (navy; n = 11), controls with matched PVLs (green; n = 12), and patients with ATL (red; n = 6). (C) Comparison of the rate at which autologous CD8**^+^** T cells kill polyclonal Tax-expressing HTLV-1–infected T cells (Tax**^+^**VβX^−^CADM1**^+^** cells). (D) The proportion of VβX**^+^**CADM1**^+^** cells killed with varying CD8**^+^** T-cells-to-target ratios was measured in high-risk carriers (navy; n = 12), controls with matched PVLs (green; n = 12); and patients with ATL (red; n = 6). (E) Plot showing the comparison of the rate at which autologous CD8**^+^** T cells kill high-PVL nonclonal cells in controls and in ATL and ATL-like clones in patients with ATL and in high-risk carriers (VβX**^+^**CADM1**^+^** cells). ATL and ATL-like clones were separated based on Tax expression levels. Horizontal line denotes median in panels C and E. Statistical analysis: Kruskal-Wallis test with Dunn post-test, 95% confidence interval. ns denotes *P* > .05; ∗, *P* < .05, and ∗∗, *P* < .01. E:T ratio, effector:target ratio.

To characterize HTLV-1–specific CD8**^+^** T-cell responses in high-risk carriers, an ex vivo CD8**^+^** T-cell killing assay was performed. CD8**^+^** T-cell–depleted PBMCs were cultured for 18 hours either alone or in the presence of autologous CD8**^+^** T cells at a range of effector-to-target ratios (n = 11 high-risk carriers, n = 12 controls, and n = 6 patients with ATL). Representative data of the CD8**^+^** T-cell killing assay from 1 patient (Figure 2A) show a reduction in frequency of live Tax**^+^**CD4**^+^** T cells as the relative frequency of CD8**^+^** T cells increases.

To identify whether CD8**^+^** T cells in each group were able to kill Tax-expressing, high-PVL nonclonal HTLV-1–infected cells, the rate at which the CD8**^+^** T cells kill Tax**^+^**VβX**^−^**CADM1**^+^** cells was quantified (Figure 2B). Examples of efficient and inefficient CD8**^+^** T-cell killing of Tax**^+^**VβX**^−^**CADM1**^+^** cells are shown in supplemental Figure 2A-B. CD8**^+^** T cells from patients with ATL had a significantly lower ability to kill autologous Tax**^+^**VβX**^−^**CADM1**^+^** cells than CD8**^+^** T cells from controls (median, 0.004 vs 0.071; Figure 2C). In addition, the rate of CD8**^+^** T-cell killing in high-risk carriers was significantly lower than the rate of CD8**^+^** T-cell killing in controls (median, 0.012 vs 0.071) but not significantly different from the rate of CD8**^+^** T-cell killing in patients with ATL.

After observation that 50% of ATL-like clones are Tax^low^, the rate of CD8**^+^** T-cell killing of VβX**^+^**CADM1**^+^** cells was quantified (Figure 2D-E). The rate of CD8**^+^** T-cell killing of both Tax^low^ ATL-like and ATL clones was significantly lower than the rate of CD8**^+^** T-cell killing of high-PVL nonclonal cells in controls (median, 0.005 vs 0.021; and median, 0.000 vs 0.021, respectively).

These results demonstrate that the killing of HTLV-infected cells by autologous CD8**^+^** T cells in high-risk carriers is significantly different from that in controls, despite a similar antigen burden.

### Frequency of Tax- and HBZ-specific IFN-γ**^+^**CD8**^+^** T cells

**Figure 3.**
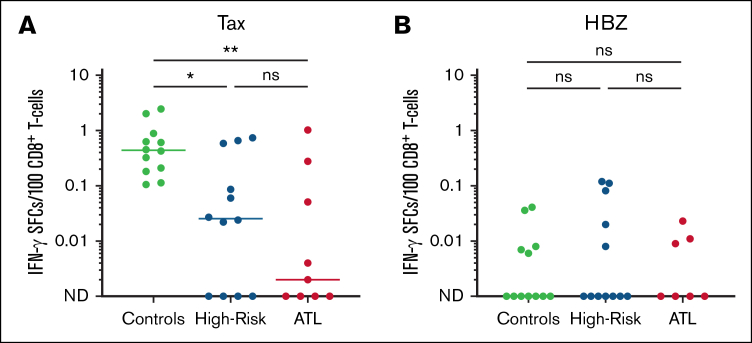
**Frequency of IFN-γ–producing CD8^+^ T cells in response to HTLV-1 antigens.** CD4**^+^** cell–depleted PBMCs of high-risk carriers (n = 12), controls with matched PVLs (n = 12), and patients with ATL (n = 9) (supplemental Table 1) were incubated for 6 hours in the presence of pools of overlapping peptides spanning the entire Tax or HBZ proteins. IFN-γ production by CD8**^+^** T cells was assayed by IFN-γ ELISpot. (A) Frequency of IFN-γ–producing CD8**^+^** T cells in response to Tax peptides. (B) Frequency of IFN-γ–producing CD8**^+^** T cells in response to HBZ peptides. Horizontal line denotes median. Statistical analysis: Kruskal-Wallis test with Dunn post-test, 95% confidence interval. ns denotes *P* > .05; ∗, *P* < .05; and ∗∗, *P* < .01. ND, not detected.

To evaluate the antigenic specificity of HTLV-1–specific CD8**^+^** T cells, we quantified the frequency of IFN-γ–producing CD8**^+^** T cells (IFN-γ**^+^**CD8**^+^** T cells) in response to overlapping peptides from Tax and HBZ in the same cohort. Tax-specific IFN-γ**^+^**CD8**^+^** T cells were detected in 100% (12/12) of controls, 66% (8/12) of high-risk carriers, and 55% (5/9) of patients with ATL (Figure 3A). The frequency of Tax-specific IFN-γ**^+^**CD8**^+^** T cells was significantly lower in patients with ATL (median = 0.002% of CD8**^+^** T cells) compared to controls (median = 0.439% of CD8**^+^** T cells). Additionally, the frequency of Tax-specific IFN-γ**^+^**CD8**^+^** T cells in high-risk carriers (median = 0.026%) was significantly lower than in controls but not significantly different from the frequency of Tax-specific IFN-γ**^+^**CD8**^+^** T cells in patients with ATL.

The frequency of HBZ-specific IFN-γ**^+^**CD8**^+^** T cells was close to the assay limit of detection. In controls, a significantly lower frequency of HBZ-specific IFN-γ**^+^**CD8**^+^** T cells compared to Tax-specific IFN-γ**^+^**CD8**^+^** T cells was detected (median = 0.000% vs 0.439%; *P* < 0.0001; Mann-Whitney, 2-tailed, 95% confidence interval). There was no significant difference in the frequency of HBZ-specific IFN-γ**^+^**CD8**^+^** T cells between the 3 groups, with HBZ-specific IFN-γ**^+^**CD8**^+^** T cells detected in 42% (5/12) of controls, 42% (5/12) of high-risk carriers, and 43% (3/7) of patients with ATL (Figure 3B).

### Analysis of IFN-γ**^+^**CD8**^+^** T cells prior to detection of an ATL-like clone

**Figure 4.**
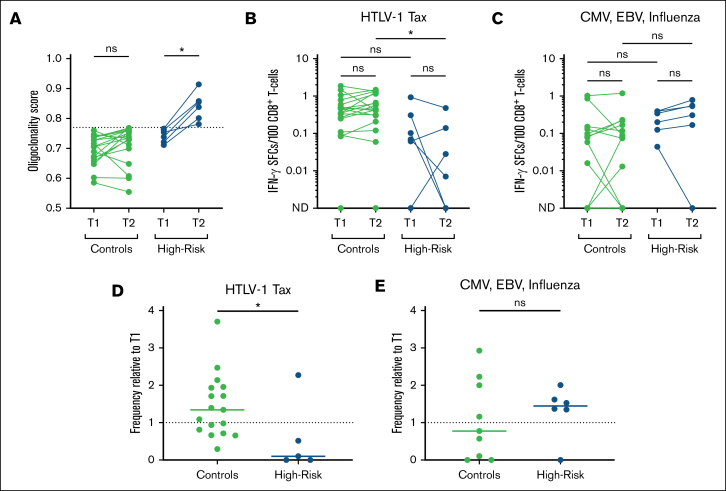
**Longitudinal analysis of the frequency of IFN-γ–producing CD8^+^ T cells.** (A) Oligoclonality score of controls and high-risk carriers at the 2 time points (T1 and T2) analyzed. High-risk carriers have an oligoclonality score above the threshold of 0.770 (dotted line) at T2. (B) Frequency of IFN-γ–producing CD8**^+^** T cells in response to pools of overlapping peptides spanning the entire Tax protein or (C) a pool of a mixture of peptides from CMV, EBV, and influenza virus (CEF). The frequency of IFN-γ–producing CD8**^+^** T cells was assayed by IFN-γ ELISpot. (D) The frequency of Tax-specific IFN-γ**^+^**CD8**^+^** T cells at T2 relative to T1 in patients with a detectable response at T1. (E) The frequency of CEF-specific IFN-γ**^+^**CD8**^+^** T cells at T2 relative to T1 in patients with a detectable response at T1. Horizontal line in panels D and E denotes median. Statistical analysis: (A) Wilcoxon matched-pairs test, 2-tailed, 95% confidence interval; (B-C) Kruskal-Wallis test with Dunn post-test, 95% confidence interval; (D-E) Mann-Whitney, 2-tailed, 95% confidence interval. ns denotes *P* > 0.05 and ∗*P* < 0.05. ND, not detected.

To investigate whether there was a relationship between HTLV-specific CD8**^+^** T cells and the emergence of an ATL-like clone, we assembled a rare series of samples from high-risk carriers before (T1) and after (T2) ATL-like clones became detectable in the blood (Figure 4A; n = 6; mean time difference, 5 years; range, 1-9). A series of PVL-matched controls were also analyzed (n = 18; mean time difference, 4 years; range, 1-8). The frequency of Tax-specific IFN-γ**^+^**CD8**^+^** T cells was quantified (Figure 4B).

The frequency of Tax-specific IFN-γ**^+^**CD8**^+^** T cells in high-risk carriers was significantly lower than in controls at T2, but not T1. There was no significant difference in the frequency of Tax-specific IFN-γ**^+^**CD8**^+^** T cells between T1 and T2 in both high-risk carriers (median T1 = 0.086% vs median T2 = 0.018% of CD8**^+^** T cells) and controls (median T1 = 0.400% vs median T2 = 0.436%) (Figure 4B). However, in 66% (4/6) of high-risk carriers, a lower frequency of Tax-specific IFN-γ**^+^**CD8**^+^** T cells was observed at T2 compared to T1. The frequency of Tax-specific IFN-γ**^+^**CD8**^+^** T cells at T2 relative to T1 was calculated in HTLV-1 carriers with a detectable response (>0.000% of CD8**^+^** T cells) at T1. High-risk carriers had a significantly lower frequency of Tax-specific IFN-γ**^+^**CD8**^+^** T cells at T2 relative to T1 (median = 0.1) than controls (median = 1.3) (Figure 4D).

To test whether the CD8 response to other viruses is also dysregulated in high-risk carriers, the frequency of IFN-γ**^+^**CD8**^+^** T cells in response to 8- to 12-mer peptides (CEF) derived from CMV, EBV, and influenza virus was also measured by ELISpot. HTLV-1 carriers were confirmed to be CMV and EBV seropositive (supplemental Table 1). The frequency of CEF-specific IFN-γ**^+^**CD8**^+^** T cells in high-risk carriers was not significantly different from that in controls at T1 and T2. There was no significant difference in the frequency of CEF-specific IFN-γ**^+^**CD8**^+^** T cells between T1 and T2 in both high-risk carriers (median at T1 = 0.272% vs median at T2 = 0.422%) and controls (median at T1 = 0.008% vs median at T2 = 0.007%) (Figure 4B). There was no significant difference in the frequency of CEF-specific IFN-γ**^+^**CD8**^+^** T cells at T2 relative to T1 in high-risk carriers (median = 1.4) compared to controls (median = 0.8) (Figure 4E).

The results suggest that a decrease in the frequency of Tax-specific IFN-γ**^+^**CD8**^+^** T cells was associated with the emergence of a detectable ATL-like clone. In addition, the results demonstrate that CD8**^+^** T cells of high-risk carriers can produce IFN-γ in response to CMV, EBV, and influenza virus peptides.

### Reduced frequency of Tax-specific CD8**^+^** T cells in high-risk carriers

To characterize the frequency and phenotype of Tax-specific CD8**^+^** T cells in high-risk carriers, we analyzed Tax_11-19_–specific CD8**^+^** T cells using HTLV-1 Tax_11-19_/HLA-A∗0201 pentamers. Tax_11-19_ peptide binds strongly to HLA-A∗0201 molecules and is an immunodominant epitope in HLA-A∗0201**^+^** HTLV-1 carriers. CMV pp65-specific CD8**^+^** T cells were also characterized using CMV pp65_495-503_/HLA-A∗0201 pentamers.

**Figure 5.**
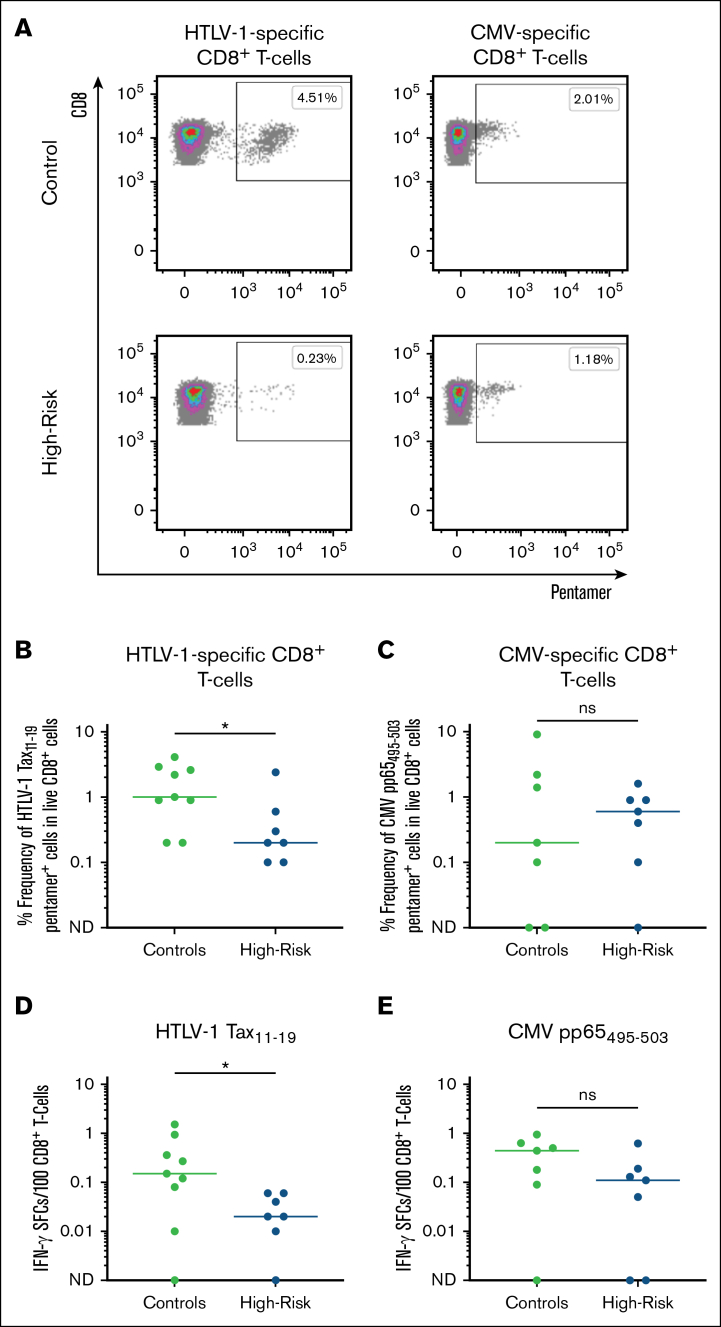
**Frequency of HTLV-1– and CMV-specific CD8^+^ T cells in HLA-A∗0201^+^ HTLV-1 carriers.** Cryopreserved PBMCs from 16 HLA-A∗0201**^+^** HTLV-1 carriers (high-risk carriers [n = 7] and controls with matched PVLs [n = 9]; supplemental Table 1) were stained with HTLV-1 Tax_11-19_/HLA-A∗0201 and CMV pp65_495-503_/HLA-A∗0201 pentamers. (A) Representative data of flow cytometry plots from a control and high-risk carrier gated on live CD3**^+^**CD8**^+^** cells. (B) Frequency of HTLV-1 Tax_11-19_/HLA-A∗0201 pentamer**^+^**CD8**^+^** cells in live CD8**^+^** T cells (high-risk [n = 7] and controls [n = 9]). (C) Frequency of CMV pp65_495-503_/HLA-A∗0201 pentamer**^+^**CD8**^+^** cells in live CD8**^+^** T cells in the CMV seropositive HTLV-1 carriers from this cohort (high-risk [n = 7] and controls [n = 7]). (D) Frequency of IFN-γ–producing CD8**^+^** T cells in response to HTLV-1 Tax_11-19_ (high-risk [n = 7] and controls [n = 9]) and (E) in response to CMV pp65_495-503_ in the CMV seropositive HTLV-1 carriers (high-risk [n = 7] and controls [n = 7]) was assayed by IFN-γ ELISpot. Horizontal line in panels B-E denotes the median. Statistical analysis: Mann-Whitney, 2-tailed; 95% confidence interval. ns denotes *P* > .05 and ∗, *P* < .05. ND, not detected.

Patients attending the clinic were screened for HLA type, and similar frequencies of each HLA type were identified in high-risk carriers and controls, with 26.7% of controls (44/165) and 24.1% of high-risk carriers (7/29) being HLA-A∗0201**^+^**. The frequency and phenotype of Tax-specific and pp65-specific CD8**^+^** T cells was characterized in 16 HLA-A∗0201**^+^** HTLV-1 carriers (n = 7 high-risk carriers; n = 9 PVL-matched controls). Representative data illustrating Tax_11-19_ pentamer**^+^**CD8**^+^** T cells and pp65_495-503_ pentamer**^+^**CD8**^+^** T cells in a control and a high-risk carrier are shown in Figure 5A. High-risk carriers had a significantly lower frequency of Tax_11-19_ pentamer**^+^**CD8**^+^** T cells (median = 0.2% of CD8**^+^** T cells) than the control group (median = 1.0% of CD8**^+^** T cells) (Figure 5B). However, there was no significant difference in the frequency of pp65_495-503_ pentamer**^+^**CD8**^+^** T cells in high-risk carriers (median = 0.6% of CD8**^+^** T cells) compared to controls (median = 0.2% of CD8**^+^** T cells) (Figure 5C).

To assess the functional capacity of the Tax_11-19_- and pp65_495-503_–specific CD8**^+^** T cells, the frequency of CD8**^+^** T cells that produced IFN-γ in response to each peptide (Tax_11-19_ and pp65_495-503)_) was characterized. Additionally, to indirectly assess the functional capacity of peptide-specific CD8**^+^** T cells in response to respective peptides, the IFN-γ spot-forming cells per pentamer**^+^**CD8**^+^** T cells (IFN-γ**^+^**pentamer**^+^**CD8**^+^** T cells) were calculated. High-risk carriers had a significantly lower frequency of HTLV-1 Tax_11-19_–specific IFN-γ**^+^**CD8**^+^** T cells compared to controls (Figure 5D); however, there was no significant difference in the frequency of HTLV-1 Tax_11-19_–specific IFN-γ**^+^**pentamer**^+^**CD8**^+^** T cells between the 2 groups (supplemental Figure 9A). Moreover, there was no significant difference in the frequency of CMV pp65_495-503_–specific IFN-γ**^+^**CD8**^+^** T cells (Figure 5E) and CMV pp65_495-503_–specific IFN-γ**^+^**pentamer**^+^**CD8**^+^** T cells (supplemental Figure 9B) in high-risk carriers and controls.

**Figure 6.**
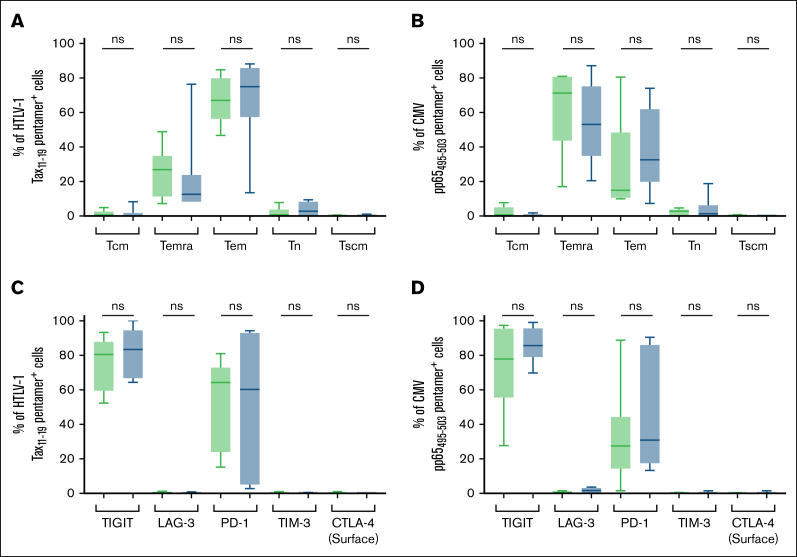
**Phenotype of HTLV-1– and CMV-specific CD8^+^ T cells in HLA-A∗0201^+^ HTLV-1 carriers.** Cryopreserved PBMCs from 16 HLA-A∗0201**^+^** HTLV-1 carriers (high-risk carriers [blue; n = 7] and controls with matched PVLs [green; n = 9]; supplemental Table 1) were stained with HTLV-1 Tax_11-19_/HLA-A∗0201 and CMV pp65_495-503_/HLA-A∗0201 pentamers, along with memory T-cell subset markers (CD45RA, CD95, CCR7) and extracellular checkpoint proteins (PD-1, TIGIT, TIM-3, CTLA-4, LAG-3). The phenotype of pp65-specific CD8**^+^** T cells was characterized in the CMV seropositive HTLV-1 carriers with detectable pp65-specific CD8**^+^** T cells (>0.0% of CD8**^+^** T cells) of the cohort (high-risk [n = 6] and controls [n = 5]). Memory T-cell subsets: stem cell (Tscm, CD95**^+^**CD45RA**^+^**CCR7**^+^**), naïve (Tn, CD95**^-^**CD45RA**^+^**CCR7**^+^**), central memory (Tcm, CD45RA^−^CCR7**^+^**), effector memory (Tem, CD45RA^−^CCR7^−^), and terminally differentiated effector memory (Temra, CD45RA**^+^**CCR7^−^). (A) Memory phenotype of HTLV-1 Tax_11-19_/HLA-A∗0201 pentamer**^+^**CD8**^+^** cells (high-risk [n = 7] and controls [n = 9]). (B) Memory phenotype of CMV pp65_495-503_/HLA-A∗0201 pentamer**^+^**CD8**^+^** cells (high-risk [n = 6] and controls [n = 5]). (C) Checkpoint proteins expressed by HTLV-1 Tax_11-19_/HLA-A∗0201 pentamer**^+^**CD8**^+^** cells (high-risk [n = 7] and controls [n = 9]). (D) Checkpoint proteins expressed by CMV pp65_495-503_/HLA-A∗0201 pentamer**^+^**CD8**^+^** cells (high-risk [n = 6] and controls [n = 5]). Error bars show minimum and maximum values. The box extends from the 25th to 75th percentile, with a line at the median. Statistical analysis: Mann-Whitney, 2-tailed; 95% confidence interval. ns denotes *P* > .05.

There was no significant difference in memory T-cell phenotype of Tax-specific and pp65-specific CD8**^+^** T cells in high-risk carriers compared to controls (Figure 6A-B). Additionally, there was no significant difference in the frequency of checkpoint control molecules (PD-1, TIGIT, TIM-3, LAG-3, and surface CTLA-4) expressed by Tax-specific and pp65-specific CD8**^+^** T cells in high-risk carriers compared to controls (Figure 6C-D).

These findings suggest that the absolute frequency of HTLV-1–specific CD8**^+^** T cells is significantly lower in high-risk carriers than in controls; however, on a per-cell basis, the phenotype and function of HTLV-1–specific CD8**^+^** T cells do not differ in high-risk carriers and controls.

## Discussion

Most HTLV-1 carriers generate high frequencies of chronically activated HTLV-1–specific CD8**^+^** T cells.^9^ However, despite the presence of HTLV-1 in the tumor, HTLV-1–specific CD8**^+^** cells are often undetectable in ATL.^14^ Little is known about the CD8**^+^** T-cell response during ATL development. The identification of high-risk HTLV-1 carriers,^22^ with detectable HTLV-1–infected ATL-like clones in their peripheral blood, provides a unique opportunity to characterize HTLV-1–specific immune responses at the premalignant stage.

The anti-Tax CD8**^+^** T-cell response is thought to be critical for immune control of HTLV-1 infection and for determining the HTLV-1 PVL set point. Tax is a key viral regulatory protein and contains highly immunogenic epitopes.^27^ Hence, by targeting and eliminating Tax-expressing cells, CD8**^+^** T cells contribute to long-term control of the HTLV-1–infected cell population. The reservoir of Tax-expressing nonclonal HTLV-1–infected cells may play a role in contributing to the survival of the expanded ATL and ATL-like clones. To minimize antigen-burden effects, we compared PBMCs from high-risk carriers with PVL-matched controls with no detectable ATL-like clones in their PBMCs. Both high-risk carriers and controls studied in this paper had a very high burden of infection. Using an in vitro killing assay, we demonstrated that autologous CD8**^+^** T cells from high-risk carriers and patients with ATL were significantly less efficient at killing Tax-expressing HTLV-1–infected cells than CD8**^+^** T cells from PVL-matched controls. In addition, there was a significantly lower frequency of Tax-specific IFN-γ**^+^**CD8**^+^** T cells in high-risk carriers and patients with ATL compared to controls. In high-risk carriers, a significantly reduced frequency of Tax-specific IFN-γ**^+^**CD8**^+^** T cells was observed after the emergence of a detectable ATL-like clone, although some patients displayed low frequencies of Tax-specific CD8**^+^** T cells before ATL-like clones were detected in circulation. Finally, using pentamer staining, we also observed a lower frequency of Tax-specific CD8**^+^** T cells in high-risk carriers. Although we observed no significant difference in the expression of checkpoint control molecules on Tax-specific CD8^+^ T cells from high-risk carriers vs controls, larger study sizes are required to conclusively rule out a causative role of exhaustion of HTLV-specific CD8^+^ T cells in high-risk carriers. However, preliminary evidence suggests that Tax-specific CD8**^+^** T cells are not functionally defective because the ratio of IFN-γ-producing to pentamer**^+^**CD8**^+^** T cells is not significantly different in high-risk carriers compared to controls. Together, our data suggest that the inefficient HTLV-1–specific CD8**^+^** T-cell response observed in high-risk carriers is likely due to the reduced frequency of HTLV-1–specific CD8**^+^** T cells in these individuals, similar to observations published in ATL.^14^

There is evidence that ATL clones evolve to escape the immune response.^15^, ^16^, ^17^ Tax-expressing ATL cells are preferentially killed by cultured autologous CD8**^+^** T cells.^7^ However, in over 50% of ATL cases, the expression of Tax protein is lost in the ATL clone.^15^ In this study, it was identified that 50% of high-risk carriers had ATL-like clones that did not express Tax protein (Tax^low^). Tax expression in ATL is lost due to genetic changes, such as mutations and deletions, and epigenetic changes, such as DNA methylation.^15^ We observed deletions in the provirus of 50% of Tax^low^ clones in high-risk carriers, similar to patients with ATL. Previous studies have shown that clones with 5’-LTR deleted proviruses are clonally expanded relative to those with intact proviruses.^28^ It is believed that the loss of Tax expression by ATL clones may be a potential immune escape mechanism. Using an in vitro killing assay, we observed that Tax^low^ ATL and ATL-like clones were significantly less efficiently killed by autologous CD8**^+^** T cells than high-PVL nonclonal cells in controls that did express Tax. However, the lack of observed killing of Tax^low^ malignant/premalignant clones in these donors may stem from the reduced ability of CD8^+^ T cells to mediate cytotoxicity against autologous HTLV-1–infected cells in general.

The anti-HBZ CD8**^+^** T-cell response also plays an important role in controlling HTLV-1 infection. MHC class I alleles that bind and present peptides of HBZ are associated with low risk of disease,^10^ and HBZ expression is constitutively maintained in malignant cells. We observed no significant difference in the frequency of HBZ-specific IFN-γ**^+^**CD8**^+^** T cells between controls, high-risk carriers, and patients with ATL. Only ∼40% of patients in each group had detectable HBZ-specific CD8**^+^** T-cell responses, likely because HBZ is poorly immunogenic and expressed at low levels.^10^^,^^29^^,^^30^ CD8**^+^** T-cell responses to HBZ and other viral proteins may also mediate immune pressure despite weaker immunogenicity.^27^ The low frequency of HBZ-specific T cells, which was close to the assay limit of detection, prevented us from further evaluating HBZ-specific CD8^+^ T cells in this study.

In this study, we demonstrated that evidence of the significant differences in the HTLV-1–specific CD8**^+^** T-cell response in high-risk carriers is not generalized, with no significant differences in the frequency of IFN-γ**^+^**CD8**^+^** T cells in response to a pool of peptides from CMV, EBV, and influenza virus compared to controls. Additionally, there was no significant difference in the frequency of CMV-specific CD8**^+^** T cells in high-risk carriers compared to controls, as seen in ATL.^14^ However, the antiviral immune response in high-risk carriers has not yet been explored extensively.

In conclusion, this is the first report of HTLV-1–specific immune dysregulation in the premalignant stage of ATL. Considering the critical role of HTLV-1–specific CD8**^+^** T-cell responses in controlling HTLV-1 infection, lack of HTLV-1–specific CD8**^+^** T cells may contribute to the development of ATL. Strategies to boost HTLV-1–specific CD8**^+^** T cells, such as adoptive T-cell therapy, may have potential as therapeutic interventions for ATL prevention; however, further investigation is required to determine clinical feasibility and efficacy.

Conflict-of-interest disclosure: The authors declare no competing financial interest.
