# Supplementary material for: Loss of HTLV-1–specific CD8+ T-cell immunity in virus carriers predisposed to adult T-cell leukemia/lymphoma
Source: Blood Neoplasia. 2026 Apr 27;3(3):100235. doi: 10.1016/j.bneo.2026.100235 (PMC13266014; doi:10.1016/j.bneo.2026.100235)
Supplement: Supplemental Figures [file BNEO_NEO-2025-001018-mmc2.pdf]

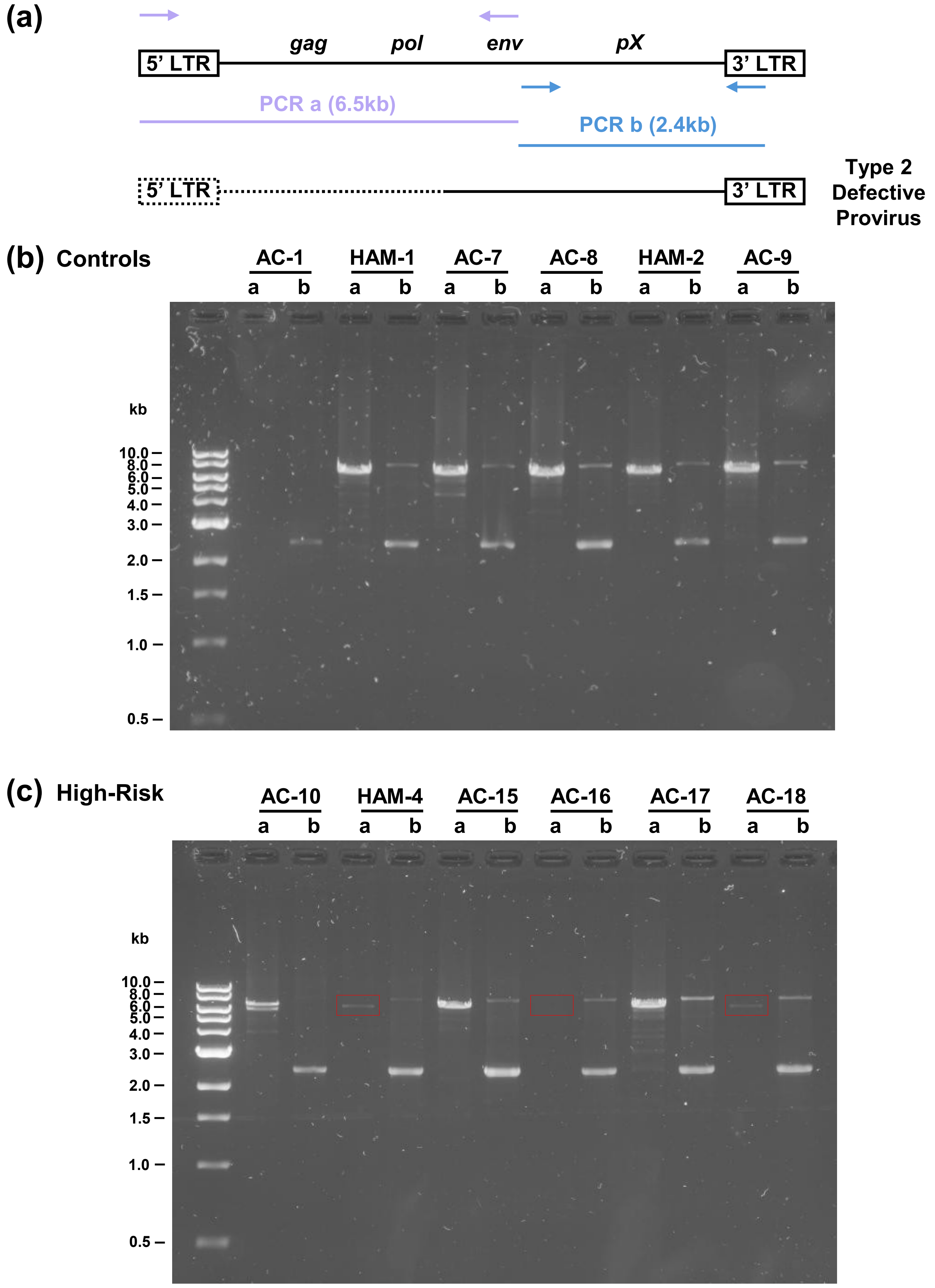

Figure S1: Determination of deletion in the provirus. (a) Schematic of primers and respective PCR products amplified from genomic DNA from patient PBMCs. In a complete provirus, both product a and product b are amplified. In a provirus with a 5' long terminal repeat (LTR) deletion, product a is not amplified, however product b is amplified. (b) Representative data from controls (n=6) with complete proviruses showing successful amplification of product a and product b. AC-1 has little amplification of both product a and b. (c) Gel of PCR products a and b of high-risk carriers with a Tax^{low} ATL-like clone. HAM-4, AC-16 and AC-17 have a faint or no band for product a (indicated by a red box) suggesting the presence of a 5' LTR deletion in the provirus of the ATL-like clone.

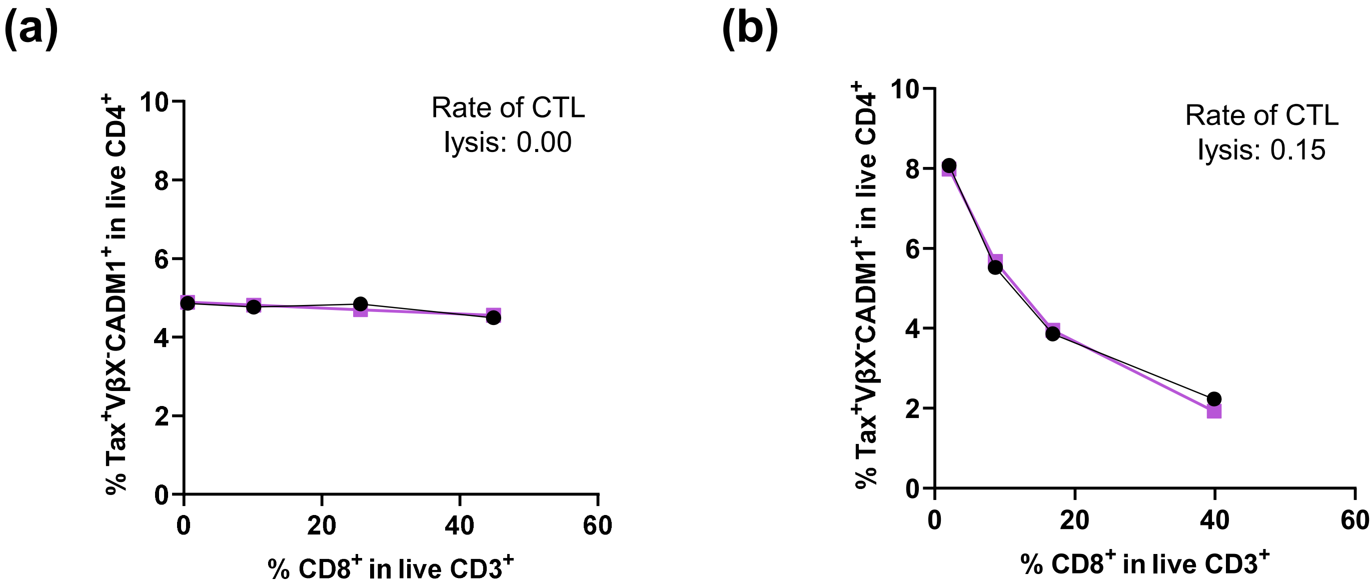

Figure S2: Estimation of the rate of $CD8^+$ T-cell-mediated lysis. $CD8^+$ cell-depleted PBMCs were incubated for 18h either alone or in the presence autologous $CD8^+$ T-cells at a range of ratios. The rate (efficiency) of $CD8^+$ T-cell-mediated lysis was then estimated by fitting the model (pink) to the experimental data (black). (a) An example of efficient and (b) inefficient $CD8^+$ T-cell-mediated lysis.

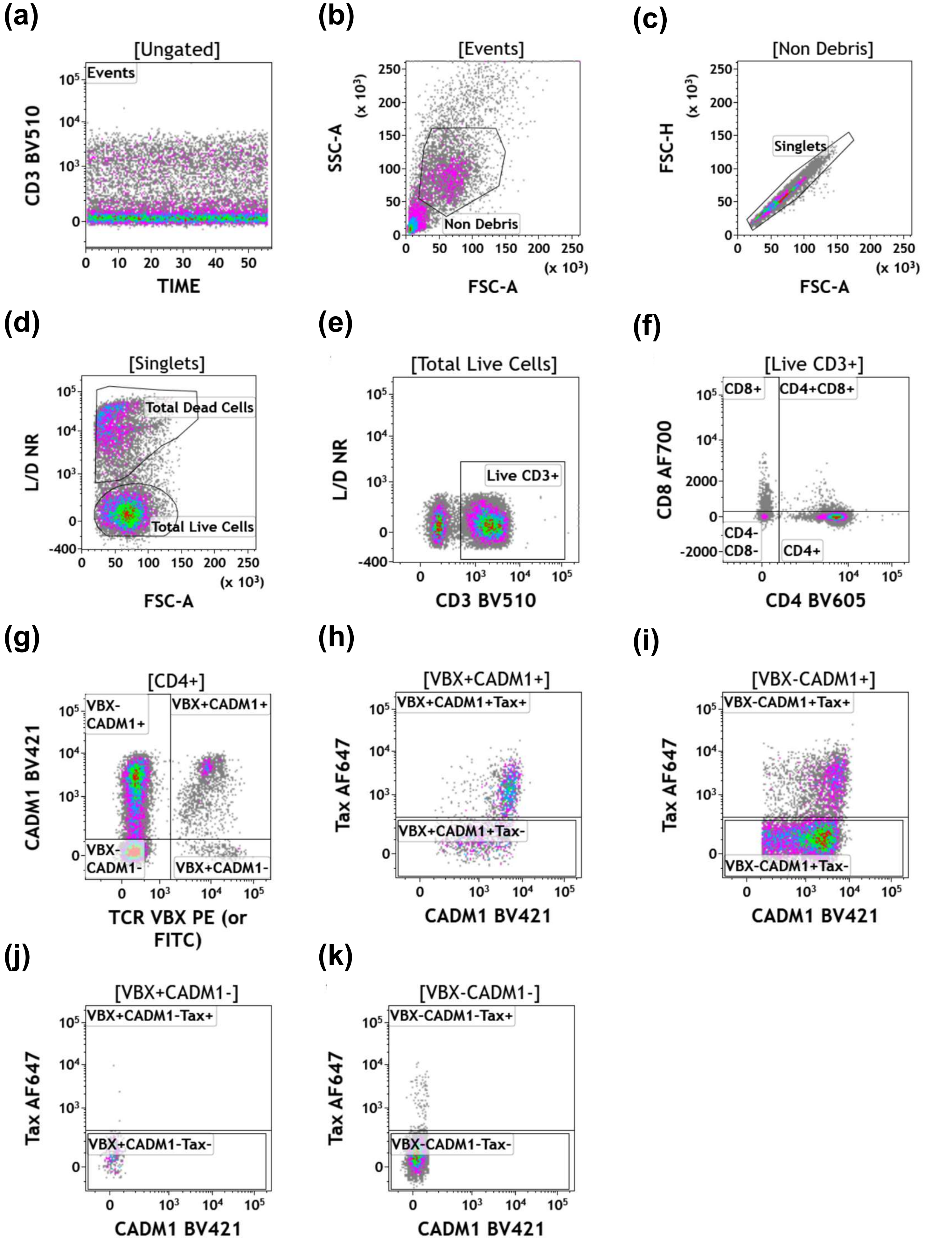

Figure S3: Gating strategy of CD8⁺ T-cell killing assay. Events acquired on the cytometer were selected (a). Non debris was then gated on the basis of forward and side scatter (b), followed by singlets (c), total live cells (d) and live CD3⁺ cells (e). Live CD3⁺ cells were then sorted into CD8⁺ and CD4⁺ cells (f). CD4⁺ cells were further sorted into (g) VβX⁺CADM1⁺ in patients with an expanded clone, VβX⁻CADM1⁺ representing polyclonal HTLV-1-infected cells, and infected cells with low proviral load/uninfected cells (VβX⁺CADM1⁻ and VβX⁻CADM1⁻). These four subsets were then sorted (h-k) into Tax-expressing (Tax⁺) and non-Tax-expressing (Tax⁻) populations.

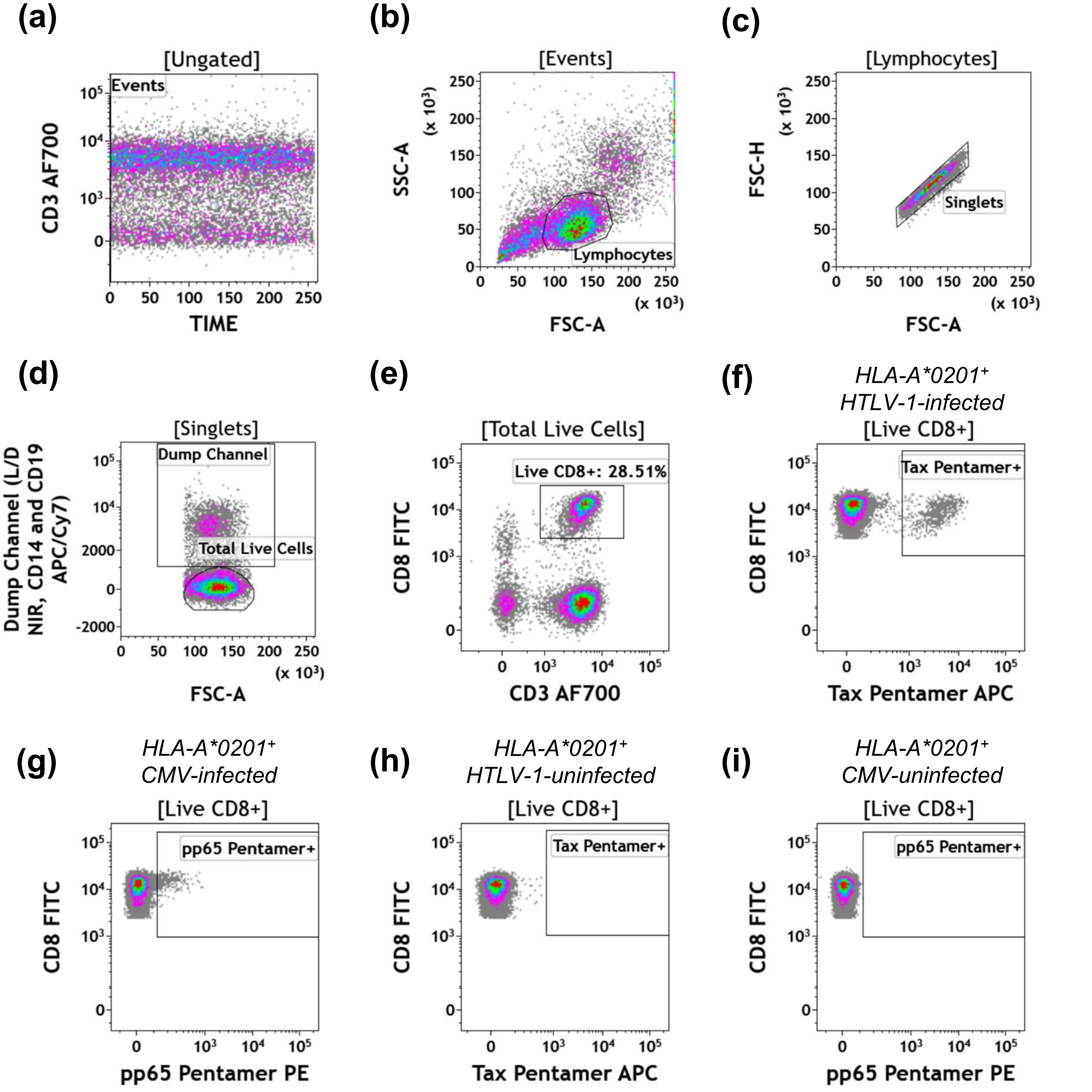

Figure S4: Gating strategy of HLA-A*0201 pentamer staining. Events acquired on the cytometer were selected (a). Lymphocytes were then gated on the basis of forward and side scatter (b), followed by singlets (c), total live cells (d) and live $CD8^+CD3^+$ cells (e). Live $CD8^+CD3^+$ cells were then sorted into HTLV-1-specific $CD8^+$ T-cells ($CD8^+Tax_{11-19}$/HLA-A*0201 $Pentamer^+$ cells) (f) and CMV-specific $CD8^+$ T-cells ($CD8^+$ $pp65_{495-503}$/HLA-A*0201 $Pentamer^+$ cells) (g). (h,i) Flow plots from an HTLV-1 and CMV uninfected donor used to select gating strategy to identify $CD8^+Tax_{11-19}$/HLA-A*0201 $Pentamer^+$ cells and $CD8^+$ $pp65_{495-503}$/HLA-A*0201 $Pentamer^+$ cells.

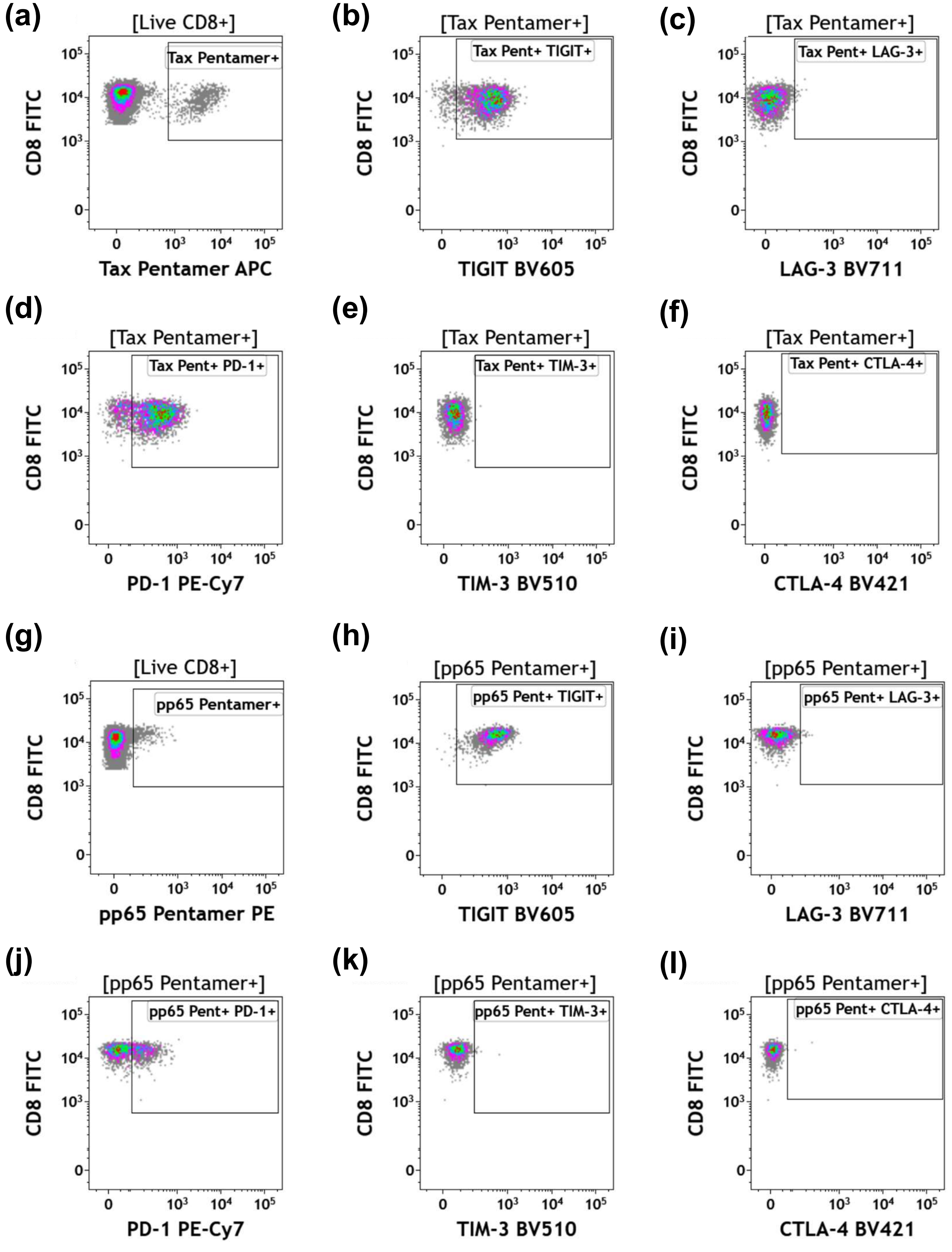

Figure S5: Gating strategy for checkpoint proteins expressed by HTLV-1- and CMV-specific CD8⁺ T-cells. PBMCs were gated as shown in supplementary figure 4 to identify HTLV-1- and CMV-specific CD8⁺ T-cells (a, g). Within these cell populations, the frequencies of cells expressing TIGIT (b, h), LAG-3 (c, i), PD-1 (d, j), TIM-3 (e, k) and CTLA-4 (f, l) was quantified.

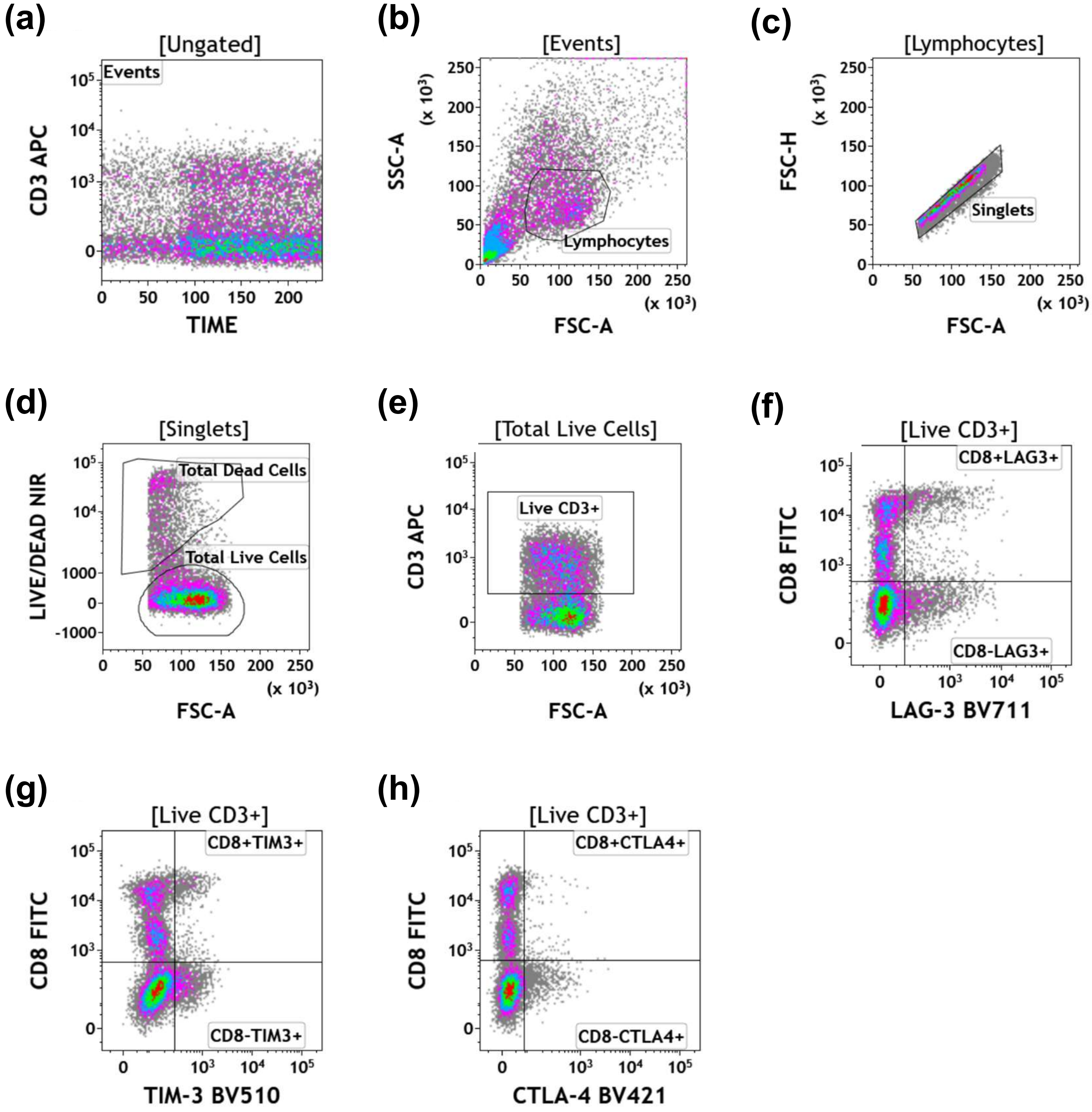

Figure S6: LAG-3, TIM-3, and CTLA-4- expression in cultured PBMCs. PBMCs from a healthy donor were stimulated with anti-CD3/anti-CD28 and cultured for 3 days to confirm that the antibodies used in the checkpoint protein staining panel could successfully detect LAG-3, TIM-3 and CTLA-4 surface markers. Events acquired on the cytometer were selected (a). Lymphocytes were then gated on the basis of forward and side scatter (b), followed by singlets (c), total live cells (d) and live $CD3^+$ cells (e). The frequency of LAG-3-expressing (f), TIM-3-expressing (g), and CTLA-4-expressing (h) $CD3^+$ cells were identified.

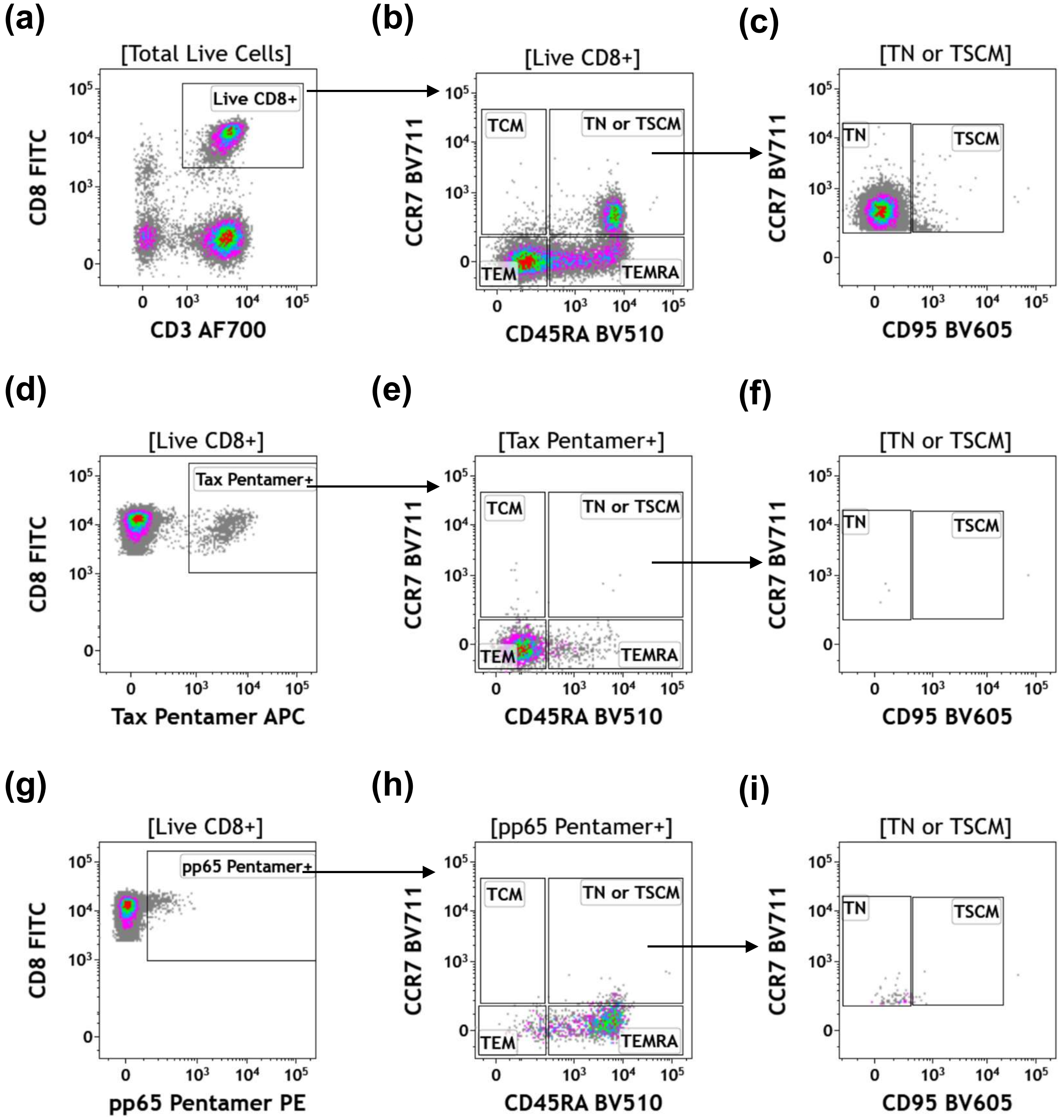

Figure S7: Gating strategy for identifying T-cell subsets of HTLV-1- and CMV-specific CD8⁺ T-cells. PBMCs were gated as shown in supplementary figure 4 to identify live CD8⁺CD3⁺ cells, HTLV-1- and CMV-specific CD8⁺ T-cells (a,d,g). These populations were then further sorted into naïve or stem cell (TN or TSCM), central memory (TCM), effector memory (TEM) and terminally differentiated effector memory (TEMRA) based on CD45RA and CCR7 expression (b,e,h). The naïve (TN) or stem cell (TSCM) were identified based on expression of CD95 (c,f,i).

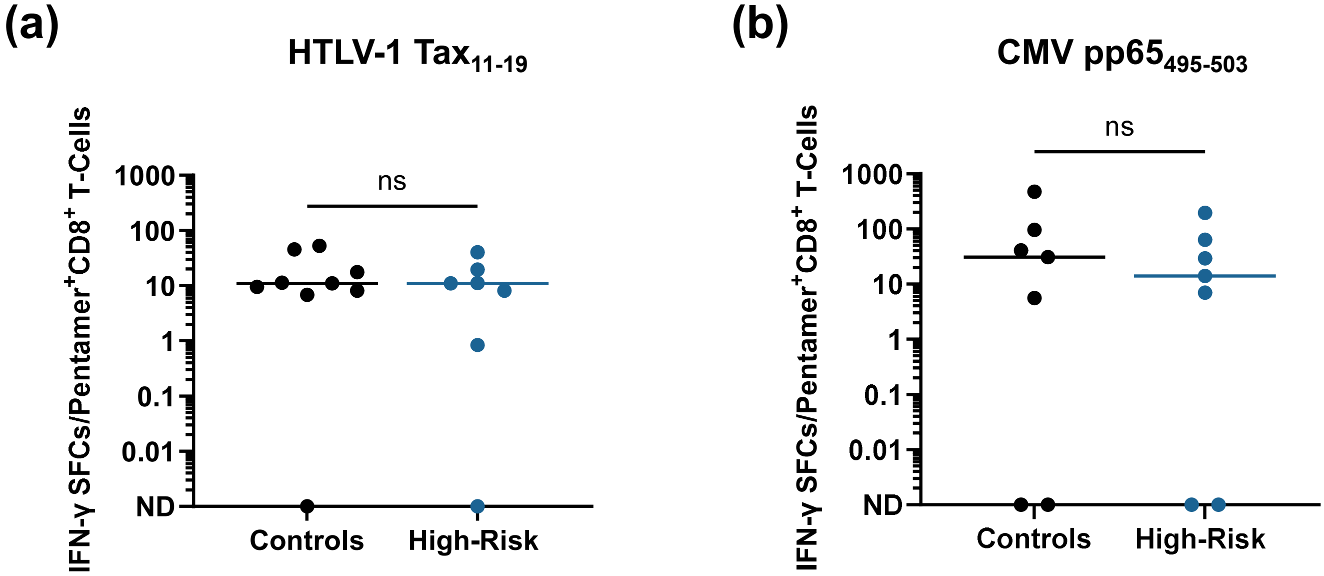

Figure S8: Frequency of IFN-γ producing pentamer$^+$CD8$^+$ T-cells in response to HTLV-1 Tax$_{11\text{-}19}$ and CMV pp65$_{495\text{-}503}$ peptide. Cryopreserved PBMCs from 16 HLA-A*0201$^+$ HTLV-1 carriers (high-risk carriers (n=7), controls with matched proviral loads (n=9) were incubated for 6h in the presence of HTLV-1 Tax$_{11\text{-}19}$ and CMV pp65$_{495\text{-}503}$ peptide. (a) Frequency of IFN-γ producing HTLV-1 Tax$_{11\text{-}19}$/HLA-A*0201 pentamer$^+$CD8$^+$ T-cells in response to HTLV-1 Tax$_{11\text{-}19}$. (b) Frequency of IFN-γ producing CMV pp65$_{495\text{-}503}$/HLA-A*0201 pentamer$^+$CD8$^+$ cells in response to CMV pp65$_{495\text{-}503}$ in the CMV seropositive HTLV-1 carriers from this cohort (high-risk (n=7) and controls (n=7)). Statistical analysis: Mann-Whitney, 2-tailed, 95% confidence interval. ns denotes p>0.05.
